# Antibacterial Effect of the Natural Polymer ε-Polylysine Against Oral Pathogens Associated with Periodontitis and Caries

**DOI:** 10.3390/polym12061218

**Published:** 2020-05-27

**Authors:** Shinechimeg Dima, Yin-Yin Lee, Ikki Watanabe, Wei-Jen Chang, Yu-Hua Pan, Nai-Chia Teng

**Affiliations:** 1 School of Dentistry, College of Oral Medicine, Taipei Medical University, 250 Wu-Hsing Street, Taipei 110, Taiwan; shinechimeg.dima@gmail.com (S.D.); innate19@hotmail.com (Y.-Y.L.); cweijen1@tmu.edu.tw (W.-J.C.); shalom.dc@msa.hinet.net (Y.-H.P.); 2 Gerodontology and Oral Rehabilitation, Graduate School of Medical and Dental Sciences, Tokyo Medical and Dental University, Tokyo 113-8510, Japan; ikki.ore@tmd.ac.jp; 3 Department of Dentistry, Shuang Ho Hospital, New Taipei 23561, Taiwan; 4 Department of Dentistry, Taipei Medical University Hospital, 250 Wu-Hsing Street, Taipei 110, Taiwan; 5 Department of Dentistry, Chang Gung Memorial Hospital, Taipei 106, Taiwan; 6 Graduate Institute of Dental & Craniofacial Science, Chang Gung University, Taoyuan 333, Taiwan; 7 School of Dentistry, College of Medicine, China Medical University, Taichung 404, Taiwan; 8 Dental Department, Taipei Medical University Hospital, Taipei 110, Taiwan

**Keywords:** polylysine, antibacterial, polypeptide

## Abstract

Antimicrobials are important adjuncts in the treatment of caries and periodontitis. However, increased bacterial resistance and hypersensitivity reactions to commonly used antimicrobials have led to an increasing demand for safe and natural substances. The objective of this study was to investigate the antibacterial effects of ε-polylysine against oral pathogens *Streptococcus mutans* and *Porphyromonas gingivalis*. Broth dilution assay, scanning electron microscopy (SEM) and confocal laser scanning microscopy (CLSM) analyses were performed to explore the antibacterial effect of ε-polylysine against *S. mutans* strain ATCC25175 and *P. gingivalis* strain ATCC332277. For the test solution, ε-polylysine was added to the bacterial suspension to prepare 0.125%, 0.25%, 0.5% and 1% ε-polylysine solutions diluted in broth medium. All four concentrations demonstrated complete inhibition of *S. mutans* and significantly reduced viable cell counts of *P. gingivalis* after 24 h. From starting inoculum of 9.15 log CFU/mL, *P. gingivalis* cell counts reduced to 4.01 log CFU/mL in the 0.125% ε-polylysine treatment group. SEM, CLSM, and the LIVE/DEAD bacterial assay of ε-polylysine application on *P. gingivalis* biofilm-dentin specimens revealed bacterial cell membrane disruption and irregular cell morphologies. The results indicated satisfactory antibacterial efficacy of ε-polylysine against *P. gingivalis* and *S. mutans* in liquid medium and as an application on biofilm-dentin specimens.

## 1. Introduction

Oral biofilms are three dimensional, dynamic microbial communities growing on the solid surfaces of teeth [1]. Dental caries and periodontitis are infectious diseases of the oral cavity, and oral biofilms are strongly associated with its etiology [2]. Dental caries is a hard tissue disease that involves acidogenic plaque bacteria, including *Streptococcus mutans*, *Streptococcus sobrinus*, and *Lactobacillus* spp. [3], residing primarily in the supragingival plaque. Periodontitis is a common chronic inflammatory disease caused by an accumulation of different pathogenic biofilm-forming bacteria in subgingival plaque, leading to an exaggerated immune response that destroys periodontal ligament and causes alveolar bone loss. *Porphyromonas gingivalis*, *Tannerella forsythia*, and *Treponema denticola* are considered to be the major pathogens involved in advancing periodontitis [3,4].

The prevention and treatment of caries and periodontitis aim to control plaque biofilms using numerous antimicrobial agents, which are formulated into oral health care products. Fluoride remains the cornerstone treatment for caries prevention; however, additional antiplaque approaches are required to enhance its effectiveness. Concurrent use of chemical substances and mechanical cleaning has been demonstrated to be beneficial in decreasing biofilm formation in periodontitis treatment. A broad-spectrum antimicrobial agent commonly used for the antiplaque approach in both caries and periodontitis treatments is chlorhexidine. Studies, however, have demonstrated increasing severe hypersensitivity reactions [5,6] and antibiotic resistance to chlorhexidine [7,8]. Although several antimicrobials, such as azithromycin [9], minocycline [10], tetracycline, and metronidazole [11], demonstrated larger improvements in periodontal health when used as local deliveries in adjunct to scaling and root planning, compared with scaling and root planning alone, adverse effects such as periodontal bacterial resistance to such antimicrobial agents have been reported [12,13,14]. The challenges caused by drug-resistant bacteria have created a need for the development of effective and safe antimicrobial compounds [15]. Ideal antibacterial compounds must be effective against a wide range of microorganisms, act rapidly, maintain activity at low concentrations, have no side effects, and be usable without discomfort.

In the past three decades, antimicrobial peptides have been researched extensively because of their antimicrobial ability and the low risk of developing bacterial resistance. ε-Polylysine is a cationic, naturally occurring polypeptide that is produced as extracellular material by *Streptomyces albulus* [16]. It was first identified by Shime and Sakai in the 1970s [17] and is produced industrially through the fermentation of *Streptomyces albulus* mutated strains for use as a food preservative. ε-Polylysine is generally regarded as a safe (GRAS) natural polypeptide consisting of L-lysine units (n = 25–30) [18] that is biodegradable, water-soluble, nontoxic, and edible. The antibacterial characteristics of ε-polylysine is well established in food industry and it is increasingly being applied in biomedicine in recent years [19,20,21,22]. Few studies have utilized ε-polylysine for dental application purposes, including composite [23,24], dental adhesive [25], implant surface modification [26] and antimicrobials [27,28]. However, the effect of ε-polylysine on *P. gingivalis* is less studied and the investigating methods of its antibacterial effectiveness against oral microorganisms were limited primarily to inhibition of planktonic bacterial growth. In addition, the demand for research on further applications of ε-polylysine in dental practice is increasing. Therefore, the objective of this study was to investigate the antibacterial effects of ε-polylysine (ε-PL) against *S. mutans* and *P. gingivalis* in planktonic growth and biofilm on the dentin surface.

## 2. Materials and Methods

### 2.1. Bacterial Strains, Growth Conditions, Culture Media, and Antimicrobial Preparation

*S. mutans* strain ATCC25175 was cultured in Tryptic soya broth and grown in Tryptic soya agar (Thermo Fischer Scientific, Waltham, MA, USA) in an anaerobic chamber with an atmosphere of 85% N_2_, 5% H_2_, and 10% CO_2_ for 2 days. A stock culture of *P. gingivalis* ATCC332277 was cultured in brain heart infusion (BHI) broth (Difco Laboratories Inc., Detroit, MI, USA) supplemented with 0.5 mg/mL hemin, 0.1 g/mL vitamin K, and 0.4 g/mL l-cysteine and used for the experiments after being assessed using gram staining. *P. gingivalis* stocks were grown in BHI agar containing 5% defibrinated sheep blood, 0.5 mg/mL hemin, 0.1 g/mL vitamin K, and 0.4 g/mL L-cysteine in an anaerobic chamber with an atmosphere of 85% N_2_, 5% H_2_, and 10% CO_2_ for 2 days. The culture temperature was maintained at 37 °C for each strain. Aliquots were stored at −70 °C. 

### 2.2. Broth Dilution Assay

The inhibitory effect of ε-polylysine against *S. mutans* and *P. gingivalis* was assessed using broth dilution assay. The initial bacterial suspension was prepared at an optical density of 0.1 using spectrophotometer (GENESYS 10S UV–Vis, Thermo Fischer Scientific, Waltham, MA, USA). The test solution, ε-polylysine, was added as the bacterial suspension to obtain ε-polylysine concentrations of 0.125%, 0.25%, 0.5%, and 1% in the broth medium. The bacterial suspension was incubated at 37 °C in anaerobic conditions. At 1, 6, 12, and 24 h, 100 µL of bacterial suspension was collected for the broth dilution assay. A serial dilution in phosphate-buffered saline (PBS) (10^1^–10^4^) was performed. Subsequently, 25 to 100 µL of bacterial suspension was inoculated on agar plates. The plates were incubated at 37 °C in an anaerobic chamber with an atmosphere of 85% N_2_, 5% H_2_, and 10% CO_2_ for 48 h, and colony-forming units (CFU/mL) were counted. Three independent experiments were performed and the mean measurements were assessed.

### 2.3. SEM Observations on Bacterial Biofilms

Recently extracted, caries-free, non-restored human third molar teeth were cleansed and stored in 0.1% thymol until use. The crowns were sectioned through cutting with a low-speed water-cooled diamond saw (Isomet; Buehler, Lake Bluff, IL, USA). The teeth were then cut along the buccolingual plane to obtain the cervical dentin for dentin specimen (4 × 4 × 1 mm^3^) preparation. The cementum was removed and the surfaces of each dentin disc were polished with 600-grit and 1000-grit silicon carbide paper under running water for 30 s each. The dentin disks were then rinsed with 1 M acetic acid for 30 s to remove the smear layer and expose dentinal tubules, rinsed with distilled water for 30 s, and then autoclaved (121 °C for 20 min). The specimens were then aseptically placed in sterile 24-well plates (Costar, Corning Life Sciences, Tewksbury, MA, USA) with the dentin side positioned upwards. A 200-μL *P. gingivalis* (ATCC 33277) bacterial suspension (approximately 10^6^ bacteria) containing 1.8 mL of BHI media supplemented with 0.1 g/mL vitamin K, 0.5 mg/mL hemin, and 0.4 g/mL L-cysteine was added to each well. The plates were incubated in anaerobic jars (37 °C) for 7 days to enable biofilm formation. The broth was changed every 2 days. All the specimens were then rinsed for 1 min (2×) in PBS (Sigma-Aldrich, St. Louis, MO, USA) to remove nonadherent bacterial cells before treatment. Biofilm-infected dentin specimens were randomly divided into five groups: negative controls (no treatment) and groups treated with ε-polylysine solutions at concentrations of 0.125%, 0.25%, 0.5%, and 1%. Four concentrations of ε-polylysine solutions were applied to the biofilm-dentin specimens using a microbrush. The dentin specimens were harvested, fixed, and mounted on aluminium stubs, before being sputter-coated with Au-Pd. Scanning electron microscopy (SU-3500, Hitachi, Tokyo, Japan) was then performed to assess biofilm inhibition. 

### 2.4. CLSM Analysis of Biofilm Inhibition

The LIVE/DEAD BacLight bacterial viability kit (Thermo Fisher Scientific, Waltham, MA, USA) was used to assess the antibacterial effect of ε-polylysine against P. gingivalis; 3 μL of each dye, SYTO 9 and propidium iodide, were added to 1 mL of distilled water to prepare the working solution. From the working solution, 200 μL was added to each of the control and treated dentin samples. The samples were incubated for 20 min at room temperature in a dark environment before confocal laser scanning microscopy (CLSM) analysis was conducted. The bacterial cells were imaged using a confocal laser microscope (Leica TCS SP5, Leica Microsystems, Wetzlar, Germany) with appropriate filters.

### 2.5. Statistical Analysis

Quantitative data are reported as means ± standard deviations. The antibacterial efficacy of ε-polylysine was assessed based on the viable cell count (log CFU/mL) after ε-polylysine treatment. Analyses of variance and Mann-Whitney tests were used to determine whether significant differences existed in terms of log CFU/mL between groups. A *p*-value of <0.05 was considered to be statistically significant.

## 3. Results

### 3.1. Antimicrobial Activity

**Figure 1:**
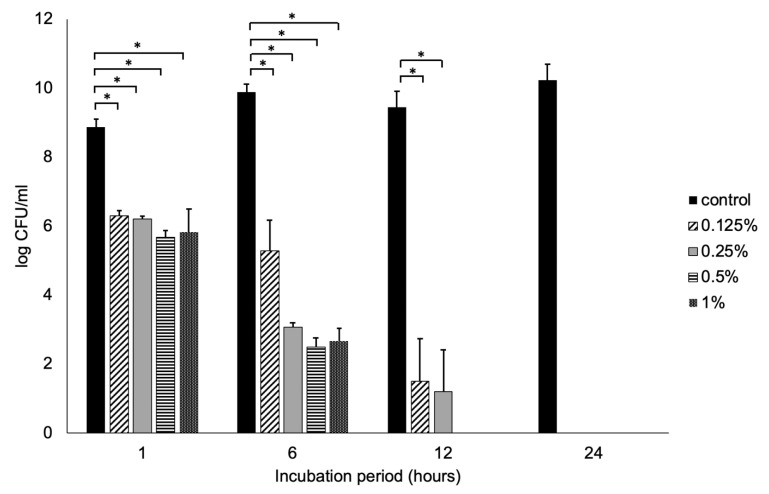
*S. mutans* bacterial viability measured as colony-forming units (CFUs) on a log scale after 0.125%, 0.25%, 0.5%, and 1% ε-polylysine treatments for 1, 6, 12, and 24 h. All values depicted are the means of triplicate measurements. * Statistical significance at *p* < 0.05 by ANOVA test.

**Figure 2:**
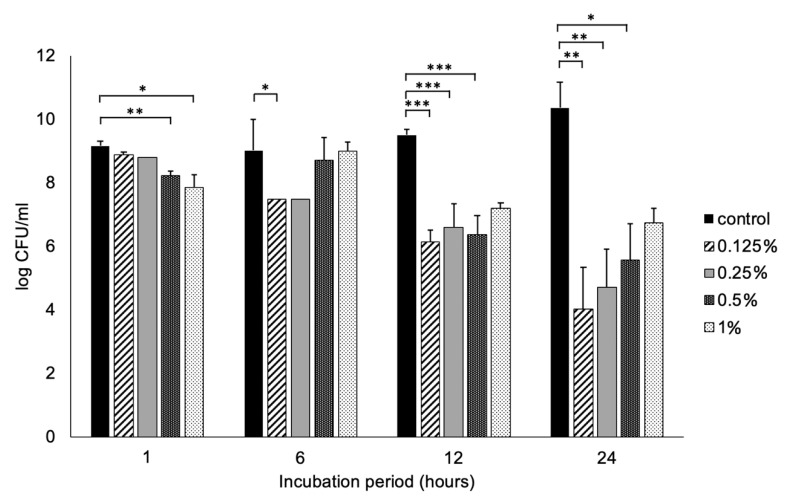
*P. gingivalis* bacterial viability measured as colony-forming units (CFU) on a log scale after 0.125%, 0.25%, 0.5%, and 1% ε-polylysine treatments for 0, 6, 12, and 24 h. All values depicted are the means of triplicate measurements. Statistical significance at * *p* < 0.05, ** *p* < 0.01, *** *p* < 0.001 by Mann-Whitney test.

Representative results from three independent experiments are displayed in Figure 1 and Figure 2. A dose-dependent response was identified in the inhibitory effect of ε-polylysine on *S. mutans* growth after 6 and 12 h. With a starting inoculum of 8.86 log CFU/mL, 0.5% and 1% ε-polylysine eliminated recoverable CFUs of *S. mutans* after 12 h (Figure 1). The viable cell counts of *S. mutans* in the 0.125% ε-polylysine group were 5.28 ± 0.88 and 1.5 ± 1.23 log CFU/ml after 6 and 12 h of incubation, respectively. The 0.25% ε-polylysine group demonstrated viable *S. mutans* cell counts of 3.07 ± 0.13 and 1.19 ± 1.22 log CFU/ml after 6 and 12 h, respectively. All four concentrations demonstrated complete inhibition against *S. mutans* after 24 h. 

The starting inoculum of *P. gingivalis* was 9.15 log CFU/ml and ε-polylysine reduced the viability of *P. gingivalis* in a time-dependent manner. The relative viability of ε-polylysine-treated cells was greater in the 1% ε-polylysine group than in the other three groups; the difference became more distinct after 24 h. Among all four treatment groups, the minimum viable *P. gingivalis* cell count was observed in 0.125% ε-polylysine group at all periods after 6 h. In 0.125% ε-polylysine treated samples, the *P. gingivalis* cell count was 6.12 ± 0.37 at 12 h which further reduced to 4.01 ± 1.31 log CFU/ml after 24 h of incubation. In contrast, the recoverable CFUs of *P. gingivalis* after 24 h of incubation was 4.69 ± 1.2, 5.56 ± 1.14 and 6.73 ± 0.45 log CFU/ml in 0.25%, 0.5%, and 1% ε-polylysine groups, respectively.

### 3.2. Inhibitory Effect on Biofilm

**Figure 3:**
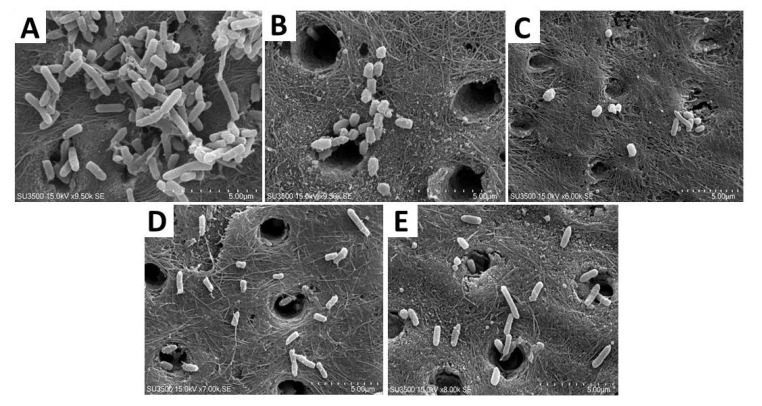
SEM images of *P. gingivalis* biofilm-dentin specimens (on day 7) after the application of different concentrations of ε-polylysine (5 µm). (**A**) control; (**B**) 0.125% ε-PL; (**C**) 0.25% ε-PL; (**D**) 0.5% ε-PL; (**E**) 1% ε-PL *P. gingivalis* bacterial cells are reduced in number and display an irregular cell morphology because of membrane disruption.

The SEM results obtained after the application of four concentrations of ε-polylysine on *P. gingivalis* biofilm-dentin disks revealed a reduced number of *P. gingivalis* bacterial cells in all four groups compared with the control group. SEM images revealed significant morphological changes in the *P. gingivalis* cells treated with ε-polylysine. The untreated cells appeared to be regular spherical shapes with smooth surfaces and intact cell walls and membranes. The SEM images of the biofilm-dentin specimens after they were exposed to ε-polylysine application revealed that *P. gingivalis* bacterial cells exhibited membrane disruption and irregular cell morphology compared with untreated cells (Figure 3).

**Figure 4:**
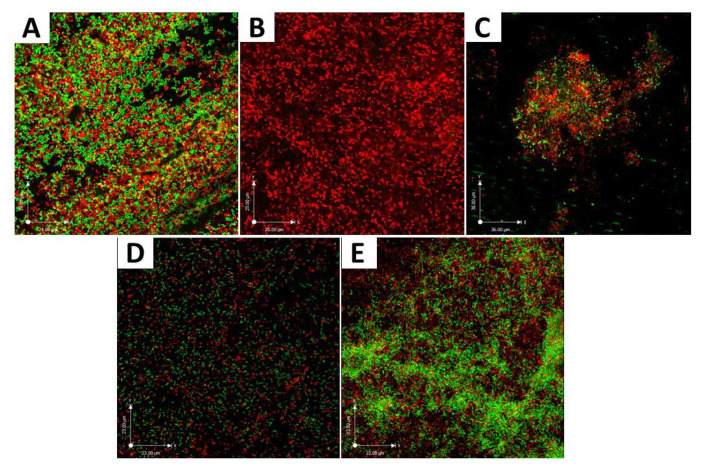
CLSM images of 7-day *P. gingivalis* biofilm on the dentin surface after application of different concentrations of ε-polylysine. Live bacterial cells are stained green (SYTO 9) and dead bacterial cells are stained red (propidium iodide). (**A**) control; (**B**) 0.125% ε-PL; (**C**) 0.25% ε-PL; (**D**) 0.5% ε-PL; (**E**) 1% ε-PL.

A CLSM experiment was performed to qualitatively assess the antibacterial effect of ε-polylysine on the *P. gingivalis* biofilm-dentin surface. The results of LIVE/DEAD bacterial staining assay after 7 days of ε-polylysine treatment on the *P. gingivalis* biofilm-dentin discs showed green and red stained bacterial cells in the control group. The 0.125% ε-polylysine treatment group had completely dead cells stained red. The 0.25% and 0.5% ε-polylysine groups presented a reduced number of live and dead bacterial cells compared with the control group. The 1% ε-polylysine group had more live cells compared than the other three experimental groups (Figure 4).

## 4. Discussion

To overcome bacterial resistance to antibiotics, studies have investigated natural antibacterial peptides because their bactericidal ability and natural derivative lower the risk of developing resistant pathogens. We investigated ε-polylysine, known for its antibacterial effects as a food preservative. To our knowledge, few studies have examined the antibacterial effect of ε-polylysine against oral pathogens. The present in vitro study investigated the antibacterial effect of ε-polylysine against oral pathogens associated with periodontitis and dental caries, *P. gingivalis* and *S. mutans*. The results demonstrated that ε-polylysine had satisfactory antibacterial effects against the tested organisms. 

We observed a significant reduction in *P. gingivalis* growth and the complete inhibition of *S. mutans* growth after 24 h of treatment with ε-polylysine. The SEM and CLSM results from the *P. gingivalis* biofilm dentin also demonstrated the damage caused by ε-polylysine to bacterial cell membranes. The antibacterial activity of ε-polylysine is attributed to the disturbance of cell membrane integrity leading to the formation of vesicles [29], oxidative stress by reactive oxygen species, and various gene expression effects [30]. The polypeptide has an affinity for bacterial membranes because of the electrostatic interaction between the negatively charged outer layer of the bacterium and the positively charged polypeptide. This attraction causes the microbes to strongly associate with these surfaces, leading to enhanced killing [29]. Moreover, the hydrophobicity of *P. gingivalis* plays an important role in its adhesion to different surfaces [31]. A previous study reported on the poly-l-lysine inhibitory effect on *P. gingivalis* fimbria binding to saliva coated hydroxyapatite [32]. 

Our results demonstrated that the susceptibility of microorganisms to ε-polylysine was dependant on ε-polylysine concentrations. The inhibitory effect of ε-polylysine against *S. mutans* increased with an increase in ε-polylysine concentration. At ε-polylysine concentrations of 0.5% and 1%, *S. mutans* was completely inhibited after 12 h, which continued until 24 h, at which point the lower concentrations of ε-polylysine, 0.125% and 0.25%, exhibited complete inhibition. Similar dose-dependent inhibitory qualities were observed in *E. coli* and *S. aureus* [33]. Dose-dependent inhibition was not observed in *P. gingivalis* in our study. In contrast to *S. mutans*, the lowest test concentration, 0.125%, was significantly more efficient in *P. gingivalis* growth inhibition compared with the higher concentrations. Numerous microorganisms are inhibited by ε-polylysine, including yeast, fungi, and Gram-positive and Gram-negative organisms [34]. Researchers who have tested the same concentrations of ε-polylysine against Gram-positive and Gram-negative organisms reported that ε-polylysine was more efficient against Gram-negative organisms if the cell membrane surface charge was considered [35,36]. Gram-negative bacteria have a higher negative charge on cell surfaces than Gram-positive bacteria do. Therefore, the interaction and improved adherence with positively charged ε-polylysine may be associated with enhanced killing. 

In this study, ε-polylysine demonstrated a higher bacterial killing effect when applied on dentin surfaces compared with bacterial killing in the liquid medium. The polypeptide binding affinity to bacterial cells was more pronounced during direct interaction with the bacterial cells compared with the bacterial suspension. Moreover, the growth medium was supplemented with various compounds that promote bacterial growth; thus, a greater bacterial killing of ε-polylysine may be observed when applied on the biofilm-dentin surface. Therefore, in clinical settings, the antibacterial effects are expected to be more effective.

## 5. Conclusions

The present study demonstrated that ε-polylysine had satisfactory antibacterial efficacy against *P. gingivalis* and *S. mutans* in the liquid culture medium and as an application on biofilm-dentin surfaces. The antibacterial effects were more pronounced against *S. mutans*. Organisms associated with periodontitis and caries may be resistant to antibiotics; thus, ε-polylysine can serve as an alternative for treating these pathogens.
